# Managing the environmental impact of research

**DOI:** 10.1186/1745-6215-12-80

**Published:** 2011-03-16

**Authors:** David C Pencheon

**Affiliations:** 1 NHS Sustainable Development Unit, Capital Park, Cambridge, UK

## Abstract

The environmental impact of research increasingly needs to be taken into account in design and execution. This makes good financial sense. However, it is especially in the research world as one of the key reasons for doing health research is to improve our knowledge to improve health. Specifically, doing research in a more sustainable way allows us to generate more knowledge with the same resource. Research not only needs to be done increasingly sustainably, but the content of the research needs to direct how we promote health and deliver healthcare in more sustainable ways.

## Introduction

Research is about reducing uncertainty so that we understand how the world works and how we can do things better: more effectively, more efficiently, and more safely. Reducing waste of any sort of resource is both an important reason for doing research and an important part of the process of conducting the research. Addressing the former is not an excuse for ignoring the latter. Research itself should therefore not be done wastefully, no matter how important we think the results might be. We should elicit maximum knowledge and understanding for the least possible investment of resource. Living in an increasingly resource constrained world only makes this more imperative. This is not just due to the current economic climate, but because of the more fundamental need for environmental and social sustainability in all we do. Research to understand how we reduce the footprint of trials is therefore especially welcome to start embedding this imperative into the practice and culture of research more widely.

## Discussion

Subaiya et al's research [1] is therefore symbolic and pioneering research and hugely important as we create a sustainable and lower carbon world. Health professionals, especially health researchers, should not consider themselves immune from either the research challenges or the research opportunities of taking the environmental impact of doing research. It is therefore increasingly important that environmental issues are systematically addressed when research is commissioned, funded, performed, disseminated and acted upon. Why?

Firstly, sustainable and low carbon research is relatively new territory, so the case needs to be made that this is important, necessary, possible, and stills delivers what we need. Studying a phenomenon is no excuse for ignoring the way it is studied. The crucial place of the research published today is that it lays some foundations for the methodologies that will be increasingly expected and routine. However these methodologies need to be continually refined in order to communicate more clearly, the costs and benefits of conducting the right research in the right way. Unless we clarify the methods for costing research more holistically, we will be ignoring the opportunities, obligations and duty we have to embed sustainability as a core part of research governance. Measures such as "Potential health gain per tonne of carbon expended" or "Patient recruited per tonne of CO_2_e" need to be tested and will become increasingly routine.

Secondly, it is important than health related research takes a lead in doing this more routinely and systematically. It would be wrong if certain disciplines thought that the importance of the potential results of research excused a blind approach to the financial, environmental and social cost of actually conducting the research. The product cannot be divorced from the process. No person, organisation or sector is immune from the challenges of a resource constrained world. Indeed, the health and health care world has much to gain from taking this seriously, and being seen to do so. Perhaps the most important reason why health researchers should take the lead is that there are just so many health co-benefits to be exploited. What is good for the health of people today is good for the health of the systems that will support us all tomorrow. Take two obvious examples. More physical activity leads to both less obesity and to a less fossil fuel dependent society. Eating less meat uses global resources more efficiently and fairly and also provides less saturated fat in our individual diets.

Thirdly, the research community has an opportunity to set an important example. A more holistic and enlightened view to the process of conducting research must be visible: just as we were reluctant to curb our smoking habits until doctors do so, similarly, society at large is unlikely to take sustainability and climate change seriously until health professionals, especially researchers, funders and journals, do the same. Fortunately, there are good examples of journals such as the British Medical Journal and the Lancet doing exactly that [2,3]. The recent publication by NIHR of carbon guidelines on sustainable research highlighted in Subaiya's article is similarly welcome.

Lastly, the academic community has a real opportunity and duty to question and study not just what we research but how we research. Trial methodology is an obvious place to start. Scrutiny and governance of research is often pioneered in this area, so it is natural that sustainable trials should become an increasingly important part of the research culture.

The future is bright only if we address our current obligations seriously and systematically. The research world is starting to do exactly that. We need to know what research to do to understand how to live more sustainably and more fairly, and we need to know how to investigate this so that the research community is part of the solution, not part of the problem. Research commissioners, funders, and doers all need to step up to the mark and design systems of sharing best practice and encouraging them to be systematically adopted and improved without increasing the bureaucracy of research. Subaiya et al's article is a welcome step on an important journey for us all. This is happening on our watch and will be our legacy.

## Conclusion

There are specific issues that need to be addressed about the environmental impacts of doing research. Most importantly is how we measure consistently and validly how we do this - what is included and what is not - and what units are measured. Secondly, to make valid comparisons, we need to have a consistent metrics to value the process (e.g. recruitment), and outcome (e.g. life years saved) of research.

Subaiya et al's article continues this important debate.

Research funders, research commissioners, and research governance systems will want to agree how best to incorporate the environmental cost well as the financial cost into the process of commissioning research.

## Competing interests

David Pencheon is Director of the NHS Sustainable Development Unit in England whose role is to shape policy to help create a healthcare system which is financially, socially, and environmentally sustainable.
